# The Cost of Leg Forces in Bipedal Locomotion: A Simple Optimization Study

**DOI:** 10.1371/journal.pone.0117384

**Published:** 2015-02-23

**Authors:** John R. Rebula, Arthur D. Kuo

**Affiliations:** 1 University of Michigan, Ann Arbor, Michigan, USA; Scientific Institute Foundation Santa Lucia, ITALY

## Abstract

Simple optimization models show that bipedal locomotion may largely be governed by the mechanical work performed by the legs, minimization of which can automatically discover walking and running gaits. Work minimization can reproduce broad aspects of human ground reaction forces, such as a double-peaked profile for walking and a single peak for running, but the predicted peaks are unrealistically high and impulsive compared to the much smoother forces produced by humans. The smoothness might be explained better by a cost for the force rather than work produced by the legs, but it is unclear what features of force might be most relevant. We therefore tested a generalized force cost that can penalize force amplitude or its *n*-th time derivative, raised to the *p*-th power (or *p*-norm), across a variety of combinations for *n* and *p*. A simple model shows that this generalized force cost only produces smoother, human-like forces if it penalizes the rate rather than amplitude of force production, and only in combination with a work cost. Such a combined objective reproduces the characteristic profiles of human walking (*R* ^2^ = 0.96) and running (*R* ^2^ = 0.92), more so than minimization of either work or force amplitude alone (*R* ^2^ = −0.79 and *R* ^2^ = 0.22, respectively, for walking). Humans might find it preferable to avoid rapid force production, which may be mechanically and physiologically costly.

## Introduction

Human locomotion is governed in part by metabolic energy economy. For a given speed, a number of aspects of an individual’s preferred gait are associated with a minimum of energetic cost [1], including the preferred stride length [2], stride width [3], and vertical displacement of the body [4], alterations to which typically lead to increased energy expenditure. However, some gait features are quite stereotypical but not obviously determined by economy, notably the ground reaction forces. Their profiles might somehow be costly to the human either metabolically or mechanically, because they must be produced by muscles and sustained by the rest of the body. But it is unclear what hypothetical costs might govern human gaits. Here we investigate possible optimization costs that might determine the force profiles produced during locomotion.

**Fig 1:**
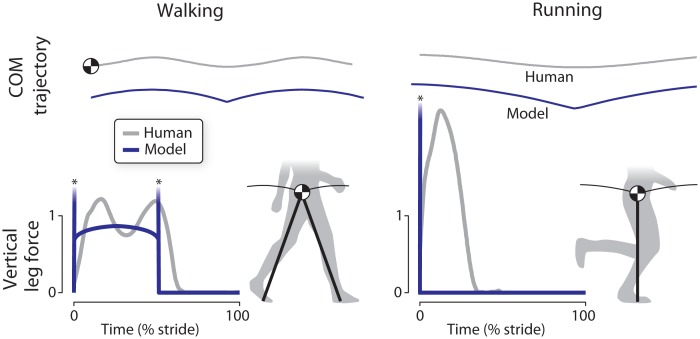
Comparison of work-minimizing model of locomotion with human walking and running gaits, in terms of body center of mass (COM) trajectories and vertical ground reaction forces vs. time. The model’s COM trajectories feature a sharp, instantaneous redirection due to ideal impulsive vertical ground reaction forces (asterisks denote infinitely high peaks, shown truncated) in both walking and running. In contrast, human gait has much smoother COM trajectories and more rounded vertical leg forces with finite peaks.

One cost in locomotion is for the production of mechanical work. During walking, work is needed to redirect the body center of mass (COM) between pendulum-like steps [5], with a roughly proportional metabolic cost [6]. A simple two dimensional walking model [7] shows that the redirection is performed most economically by applying perfectly impulsive (i.e., instantaneously brief but with high amplitude) forces along the two legs, pushing off with the back leg just before the front leg impacts the ground. A more general model relaxes the pendulum assumption and allows for arbitrary leg forces, yet still discovers the same walking gait using work minimization alone [8]. For higher speeds, the same optimization also discovers running gaits, where impulsive leg forces are again favored, except separated by aerial phases. These optimizations suggest that the strategy for reducing mechanical work would be to attempt to walk and run with rigid legs (Fig. 1).

In practice, human legs do not behave quite so rigidly. The ground reaction forces stereotypically exhibit two peaks that abstractly resemble the impulses predicted to minimize work, except with considerable smoothing (Fig. 1). Ideal impulses can theoretically be economical if they act pseudo-elastically to redirect the COM velocity with zero displacement of the actuator and therefore minimal work [5]. The relatively smoother forces produced by humans [9] would therefore be expected to perform more than the minimum possible work. This suggests the possibility that humans avoid other costs in addition to work.

A possible cost of locomotion is for active production of forces in the leg. Optimization studies have long included costs for force production to explain a variety of human motor tasks. For isometric tasks, the time-integral of muscle force [10] is associated with metabolic energy expenditure. Alternatives are to integrate the muscle force or activation, or the joint torque, raised to the second (or higher) power [11, 12], which tends to promote sharing of force between muscles (e.g., [13]). The relation to physiology is less clear, but could be associated with recruitment of less efficient motor units within a muscle as overall force increases [14, 15]. High forces might also be costly in terms of motor performance rather than economy, for example due to “signal-dependent noise” that increases movement uncertainty with force [16]. At the extreme, it may be favorable to minimize the peak forces, equivalent to a minimax optimization (e.g., [13]). Whatever the physiological justification, force costs are often required in optimization studies for practical reasons. Without them, the optimum may call for unrealistically high force application (or “bang bang control”, [17]).

A number of studies have proposed costs for high rates of force production. Human reaching tasks using the arms appear to be governed by a cost for the third derivative of position (kinematic “jerk”, [18]). A kinetic analogue to jerk is a cost for the rate of change of force or torque [19]. In the context of reaching, these and other formulations have been applied widely (e.g., [20, 21]). It is possible that such force costs could also apply to locomotion, perhaps for motor planning or because they exact a metabolic cost [22, 23]. Indeed, some proposed models of metabolic cost include multiple mechanisms related to force production (e.g. [24–27]). Such models can produce more human-like gaits than the idealized simple models [8], but it is unclear which of their features explain smoother optimal forces, and whether similar features actually govern the smooth forces of human locomotion.

In the present study, we apply a generalized model of force costs to the optimization of bipedal walking and running. We use a very simple computational model starting with a cost for mechanical work, similar to that of Srinivasan & Ruina [8]. To that objective is added the generalized force cost, which can be parametrically adjusted to model a number of the costs previously proposed in the literature. We also apply an adjustable weighting between work and the generalized force cost. We seek to determine whether some force-like costs might help explain human gaits. It is possible that a relatively simple addition to the model of Srinivasan & Ruina could better explain the ground reaction forces observed in actual walking and running, compared to the cost for mechanical work alone.

## Methods

**Fig 2:**
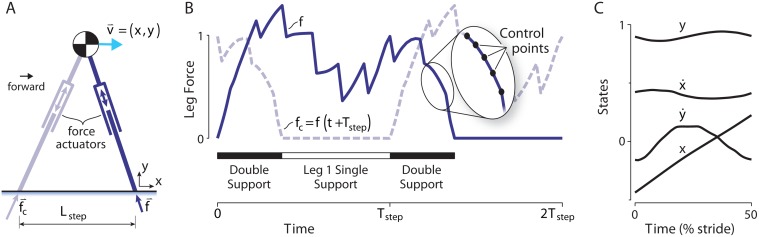
Optimization model for bipedal locomotion. (A) The model has a point mass body and massless, telescoping legs driven by axial force actuators. (B) Force magnitudes (*f* and *f*_*c*_ for the two legs) are represented as piecewise linear functions of time within a step (defined by control points, magnified in inset). (C) The state vector includes body positions and velocities (*x*, *y*, *ẋ*, *ẏ*), which result from the leg forces and equations of motion.

We formulated a computational optimization to produce periodic, bipedal locomotion (Fig. 2). The optimization objective includes a combination of mechanical work and a generalized force production cost. Optimal gaits are produced for a simple planar locomotion model, where the body is treated as a point mass, roughly approximating the location of the human body’s center of mass, about which rotate two massless legs, as in the model of Srinivasan & Ruina [8]. The legs can actively telescope and exert arbitrary commanded axial forces over time, directed along the leg axis, which extends from a foot contact point on the ground to the pelvis. This model is intended to allow for a wide range of possible center of mass trajectories and a wide variety of gaits, such as walking and running, all determined by the optimization procedure. There are a small number of constraints defined primarily to enforce steady, periodic gait with left-right symmetry. For comparison with experimental data, we define two nominal gaits representative of human locomotion: walking at a speed of 1.25 m/s and step length of 0.68 m, and running at 5.0 m/s and step length of 1.5 m (see S1 Appendix).

The optimization determines two leg force trajectories *f*(*t*) and *f*_*c*_(*t*) (contralateral leg force) over a step to minimize an objective *J*. We define that objective as a tradeoff between the proposed work and force costs, *J_w_* and *J_f_*, respectively:

$$\begin{array}{c} J=\left(1-\alpha \right)J_{w}+\alpha J_{f} \end{array}$$ (1)

where *α* is a weighting parameter ranging from 0 to 1. The value *α* = 0 denotes minimization of work alone, and should yield impulsive walking or running [8]. In contrast, *α* = 1 denotes minimization of the force cost alone. At intermediate *α* values, we expect to see the effect of combining the work and force costs.

We define the work cost as the integral of the rectified mechanical power of both legs over a step [8]:

$$\begin{array}{c} J_{w}=\int_{0}^{T_{\mathrm{step}}}\left(\left|\vec{v}\left(t\right)\cdot \vec{f}\left(t\right)\right|+\left|\vec{v}\left(t\right)\cdot \vec{f_{c}}\left(t\right)\right|\right)dt \end{array}$$ (2)

where the leg force ($\vec{f}(t)$, and ${\vec{f}}_{c}\left(t\right)$ for the contralateral leg) is directed axially along the leg. The absolute value places a positive cost on both positive and negative work. The two have different physiological costs [6], but any constant ratio between them is equivalent to an adjustment in the weighting *α*, thereby obviating the need for an additional parameter for that ratio. (See S1 Appendix for implementation details.)

The other component is the generalized force cost. We define this cost as the *p*-norm of the *n*-th derivative of force:

$$\begin{array}{c} J_{f}=\left|\right|f^{\left(n\right)}{\left|\right|}_{p}≔\sqrt[{p}]{\int_{0}^{T_{\mathrm{step}}}\left({\left|f^{\left(n\right)}\left(t\right)\right|}^{p}+{\left|f_{c}^{\left(n\right)}\left(t\right)\right|}^{p}\right)dt} \end{array}$$ (3)

where *f* ^(*n*)^(*t*) denotes the *n* ^th^ derivative of force amplitude. We will investigate *n* = [0, 1, 2], where *n* = 0 penalizes force amplitude, and greater values of *n* increase the penalty on higher-order rates of change in force. We also investigate a range of *p*, where greater values penalize larger peaks in the force cost. This cost is intended to approximately model a number of costs previously proposed in the literature, albeit in simplified form. Although detailed models of low level muscle energetics have been proposed [15, 28, 29], simple models might be helpful for conceptual exploration of the trade-offs governing locomotion. The simplifications allow investigation of various aspects of force cost and their potential effects on gait, independent of the particulars of a more complex model.

The optimization searches over trajectories of leg forces and body states to find a gait. A minimal representation of a gait comprises two leg force trajectories over one locomotion step (with duration *T*_step_), and a set of initial conditions for the periodic cycle (i.e. a fixed point of a limit cycle [30]). The body states are defined as the forward and vertical positions of the point mass pelvis (*x* and *y*, respectively) and their time-derivatives (velocities *ẋ* and *ẏ*, respectively). The relative positions of pelvis and feet determine the direction of leg forces (making the equations of motion nonlinear), and each leg force magnitude trajectory (*f* and *f*_*c*_) is described by a piecewise linear function between *N* control points, describing the axial force between pelvis and foot. The force trajectories are for one step of locomotion, equivalent to half a symmetric stride (the time between successive heelstrikes of a single leg). When both feet are on the ground, they are separated by the given step length *L*_step_. When one foot is on the ground, the swing leg is instantaneously advanced forward by one step length, after which the two leg forces are swapped to produce the second step of a stride. Although we do not model swing leg motion here, it is a simple matter to obtain arbitrary step frequencies by tuning a hip spring driving pendulum-like swing dynamics [31]. We pose no constraints on symmetry in time, but because the dynamics and cost function are time-symmetric, the optimization discovers symmetric profiles, invariant to reversal of time. Such symmetry is common to optimizing locomotion using various cost functions [32]. All state variables and other quantities are nondimensionalized using pelvis mass *M*, a maximum allowable leg length *L*, and gravitational acceleration *g* as base units. The optimization is performed using a standard MATLAB implementation of sequential quadratic programming [33].

Several constraints are applied to ensure proper gaits. The states must obey the differential equations of motion for the body with the leg forces as input. A maximum limit is also enforced on leg length, measured as the distance between pelvis and a foot on the ground. The leg’s force trajectory is constrained to zero at the beginning and end of the stride to guarantee a minimum (and possibly very brief) duration for a swing phase. To enforce continuity, leg force at the end of a step is constrained to be equal to the other leg’s force at the beginning of its step. To ensure steady locomotion at the nominal speed, the pelvis position and velocity at the end of each step are constrained to be equal to the initial pelvis state translated forward by the nominal step length, with a duration appropriate for the nominal speed. There are no features explicitly specifying walking or running gaits, which must therefore emerge from the optimization alone, as a function of the given movement speed and step length. Details of the constraints and dynamics are provided in S1 Appendix.

Optimized gaits are compared to human locomotion as a function of the cost parameters. Optimizations were performed across a range of weighting (*α*) and cost parameters (*p* and *n*), for the nominal walking and running speeds and step lengths, to yield optimal force profiles, from which three types of outcomes could be compared. First, we calculate a quantitative measure of the overall resemblance between profiles, using the correlation coefficient *R* ^2^ between ground reaction forces from model and human data (see S1 Appendix). Second, we examine the duty factor, defined as the time each leg contacts the ground, as a fraction of a stride, as well as peak force magnitudes, in comparison with human locomotion. Third, we examine the relationship between vertical leg force and vertical body displacement during stance, a relationship that is roughly linear in both walking and running gaits for a variety of animals [34]. We fit a line to calculate an effective vertical stiffness, which summarizes the overall oscillatory behavior as produced by active muscles and passive tendons. These various measures help to quantify the overall similarity between model and human.

## Results

**Fig 3:**
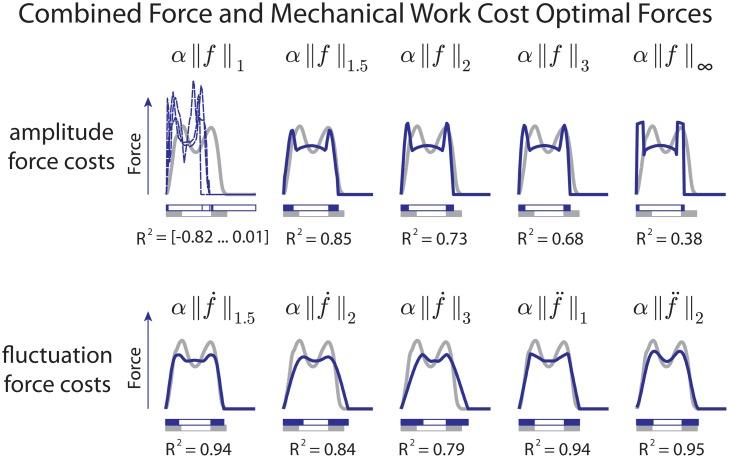
Vertical ground reaction force trajectories for various formulations of force cost *J*_*f*_ (Equation 3). Examples show the effect of varying the derivative order *n* and norm exponent *p*. Values for *α* were chosen to qualitatively match human forces where possible. The values for *α* in the cost function are (left to right), for amplitude force costs: [4.5 ⋅ 10^−1^, 5.0 ⋅ 10^−1^, 5.5 ⋅ 10^−1^], 5.0 ⋅ 10^−2^, 4.0 ⋅ 10^−2^, 4.5 ⋅ 10^−2^, and 2.0 ⋅ 10^−2^, and for fluctuation force costs: 2.7 ⋅ 10^−3^, 1.5 ⋅ 10^−2^, 2.3 ⋅ 10^−2^, 1.1 ⋅ 10^−4^, and 1.6 ⋅ 10^−3^.

The optimization yields a wide range of optimal gaits that varied considerably across cost functions considered. A general trend was that, compared to a cost for work alone, the addition of a cost for force production tended to reduce peak forces during locomotion (Fig. 3). Starting with the slower nominal speed, the optimization generally discovered walking gaits (with a ground duty factor greater than 0.5). Costs for force alone tended to be less realistic than in combination with work, and so we initially examine those combinations that best resembled human (i.e. choosing weighting *α* to yield high *R* ^2^). In these combinations, the force cost caused a reduction in the peaks produced by the work cost alone, for virtually all parameter values. Costs on force amplitude (*n* = 1) did not, however, cause force profiles to become smoother. This was only produced by costs for force fluctuations (*n* > 1), which in some cases produced reasonable resemblance to human (e.g., *R* ^2^ = 0.95 for *n* = 2, *p* = 2).

We next examine sensitivity of these results to the two cost parameters. Starting with amplitude force costs (Fig. 3, top row, *n* = 1). An increasing norm exponent *p* generally caused a reduction in the two force peaks, with a corresponding increase in force throughout the rest of stance, as is necessary for supporting body weight. There was one indeterminate case for optimization of the absolute value of force amplitude (*n* = 0, *p* = 1), which tended not to converge to a single solution, regardless of the weighting *α*. This suggests the need for higher exponents (*p* > 1) to penalize large amplitudes. Indeed, these yielded more determinate optimizations, with finite, double-peaked forces. In the limit (*p* = ∞), the cost penalizes only the maximal force, resulting in an approximation of inverted pendulum walking, with brief square wave forces replacing the instantaneous collision and pushoff forces. But as stated above, none of these combinations resulted in rounded force profiles.

In contrast, force fluctuation costs yielded better resemblance to human (Fig. 3, bottom row, *n* ≥ 1). Including a time-derivative of force (with *n* = 1 or 2), the optimization yielded somewhat differing force profiles (Fig. 3, “fluctuation force costs”) than force amplitude costs, characterized mainly by more rounded profiles. Force-derivative costs tended to be less sensitive to parameter variations in terms of peak force, force profile, and double support period. As with the force amplitude cost, an increasing norm *p* also resulted in lower peak forces, but with much subtler effects. For each parameter combination, the most human-like forces were produced with somewhat different intermediate values of *α*. Several of the parameter combinations yielded quite good matching with human data. For example, combinations (*n*, *p*) of (1.5, 1), (2, 2) and 1, 2) all yielded *R* ^2^ > 0.9.

**Fig 4:**
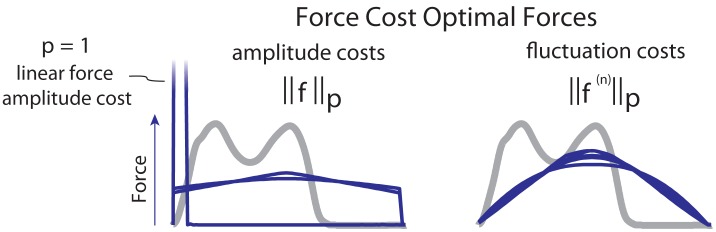
Vertical ground reaction forces for gaits optimized only for force cost. For amplitude costs (*n* = 0, left), the optimized forces resemble square waves or an instantaneous spike. For fluctuation costs (*n* > 0, right), forces smoothly rise from zero to a peak, then return to zero smoothly. All gaits (except a linear amplitude cost) exert leg forces for both legs throughout the stride except for instantaneous swing phases. In general, forces that optimize costs for force amplitude exhibit force discontinuities, while forces optimal for fluctuation costs have no discontinuities.

More insight about these effects may be gained by examining the force cost alone. Optimizing only for force cost without work revealed two broad classes of solutions for the slow nominal speed (Fig. 4). Penalizing force amplitude alone yielded force profiles with approximately instantaneous jumps in force (Fig. 4, “amplitude force costs”). In particular, penalizing only the time-integral of force magnitude (*p* = 1) yielded a single instantaneous force impulse, equivalent to the work-optimal running gait [8]. With greater exponents *p*, the optimal profile approximately resembled a square wave lasting the entire stride. This implies an instantaneously short swing period, with both feet on the ground producing force for essentially the entire gait cycle. These also included jump discontinuities in force at the beginning and end of the stride. In contrast, optimizing for force fluctuation costs alone (Fig. 4, “fluctuation costs”) produced smoothly changing forces throughout the stride, with no jump discontinuities. This qualitative difference between amplitude and fluctuation costs illustrates the source of the qualitative difference between the combined force and work costs, as the jump discontinuities are observed in the combined optimized results (Fig. 3). As force fluctuation costs were generally better able to reproduce human walking forces (based on *R* ^2^ of model-data agreement) we will consider only force fluctuation costs in further analysis, in particular, *p* = 2, *n* = 2.

**Fig 5:**
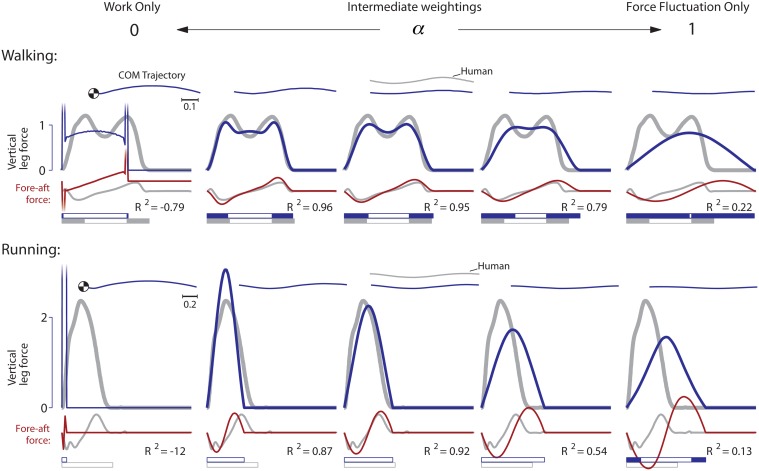
Optimized walking and running gaits as a function of weighting *α*. Shown are COM trajectories, vertical leg forces vs. time, fore-aft leg forces vs. time, and ground contact periods. Work-minimizing gaits (left side, *α* = 0) approach impulsive walking (top) and running (bottom), while force fluctuation minimizing gaits (right side, *α* = 1) have very smooth leg forces. Intermediate combinations of the two costs result in gaits with more human-like attributes, both in leg forces and in COM trajectories (shown in gray for human). Trajectories shown here are for cost parameters *n* = 2 and *p* = 2. Values for *α* for walking are (left to right): 0, 2.68 ⋅ 10^−4^, 1.61 ⋅ 10^−3^, 2.68 ⋅ 10^−3^, and 1. Values for running are: 0, 2.15 ⋅ 10^−3^, 8.05 ⋅ 10^−3^, 5.37 ⋅ 10^−2^, and 1.

We next examine the effect of variations in the weighting between work and force fluctuation costs. Minimization of work alone (*α* = 0) reproduced the results from Srinivasan & Ruina [8], yielding ideal inverted pendulum walking (Fig. 5, *α* = 0). This was characterized by rigid legs that performed work only with an impulsive collision at the beginning of a step, and a perfectly impulsive push-off at the end, with a leg duty factor approaching 0.5, so that double support approached zero duration, as predicted by other models [7].

At the other extreme, minimization of force fluctuation alone yielded an extremely smooth walking gait (Fig. 5, *α* = 1). This caused the pelvis to follow a nearly level path, with both feet on the ground for nearly the entire step. The leg duty factor approached unity, so that double support phases were separated only by infinitesimally brief single support phases to advance the swing leg. A relatively long stance phase allows the leg forces to grow and decay smoothly, resulting in relatively large leg displacements compared to the work-minimal gait.

**Fig 6:**
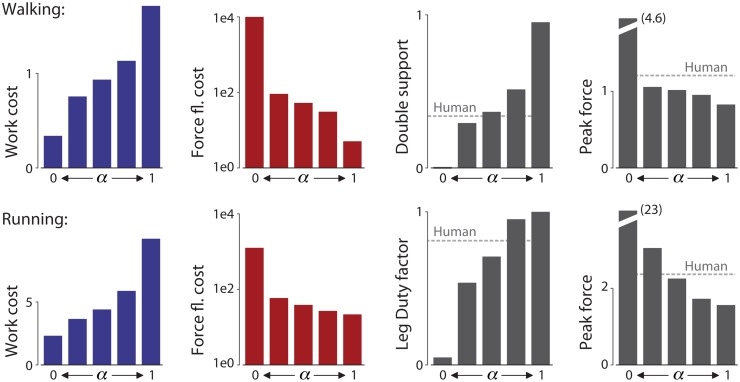
Summary measures of optimized gaits as a function of weighting *α*: Work cost, force fluctuation (fl.) cost, double support time (for walking) or leg duty factor (or ground contact time, for running) as fraction of stride, and peak vertical force. As the optimization cost function is changed from work only (*α* = 0) to force fluctuation only (*α* = 1), the work cost increases and force fluctuation cost decreases, both monotonically. The resulting double support time and leg duty factor (ground contact time) increase, and peak force decreases. Values for human (shown as dotted lines) are between the extremes found at work-minimal and force fluctuation minimal gaits.

With an intermediate weighting of force fluctuation and work costs, the walking optimization yielded more human-like leg forces. These were characterized by a smoothed double peak in vertical ground reaction force, finite double and single support durations, and intermediate force magnitudes (Fig. 5, *α* = 1.61 ⋅ 10^−3^). The weighting *α* also caused a smooth transition between two extremes. Recall that a weighting on work alone tended toward a leg duty factor of 0.5 and perfectly impulsive forces, whereas minimization of force fluctuation alone tended toward a duty factor of 1 and maximally smooth forces with low amplitude. As weighting *α* increased from zero, the two impulsive peaks in force decreased in amplitude and lengthened in duration, yielding two rounded profiles. Increasing *α* further, the two peaks combined into a single rounded peak. Increasing *α* also caused the work cost *J*_*w*_ to increase monotonically, just as the force fluctuation cost *J*_*f*_ decreased (Fig. 6). Concurrently, the duty factor increased and the peak force magnitude decreased, both monotonically. The correlation with human ground reaction forces reached a maximum of *R* ^2^ = 0.96 at an intermediate weight of *α* = 2.68 ⋅ 10^−4^. This exceeds values of *R* ^2^ = 0.79 for work only, and *R* ^2^ = 0.22 for force fluctuation only.

The optimization also yielded human-like forces for running. Minimization of work alone resulted in an impulsive grounded running gait with an infinitesimally brief ground contact, similar to that of Srinivasan & Ruina [8] (Fig. 5). In contrast, minimization of force fluctuation alone produced a very smooth gait with the pelvis following a nearly level path. As the weighting increased, the force profile remained single-peaked but became longer in duration and lower in amplitude (Fig. 6). Near unity weighting, the aerial phase disappeared, as the duty factor exceeded 0.5 and yielded a brief double support period. An intermediate weight (*α* = 8.05 ⋅ 10^−3^) resulted in a more human-like running gait with finite contact and flight phases. The correlation with human ground reaction forces reached a maximum of *R* ^2^ = 0.92 with *α* = 8.05 ⋅ 10^−3^. This exceeds values of *R* ^2^ = −12.0 for the optimization minimizing work only (*α* = 0), and *R* ^2^ = 0.13 for force fluctuations only (*α* = 1).

**Fig 7:**
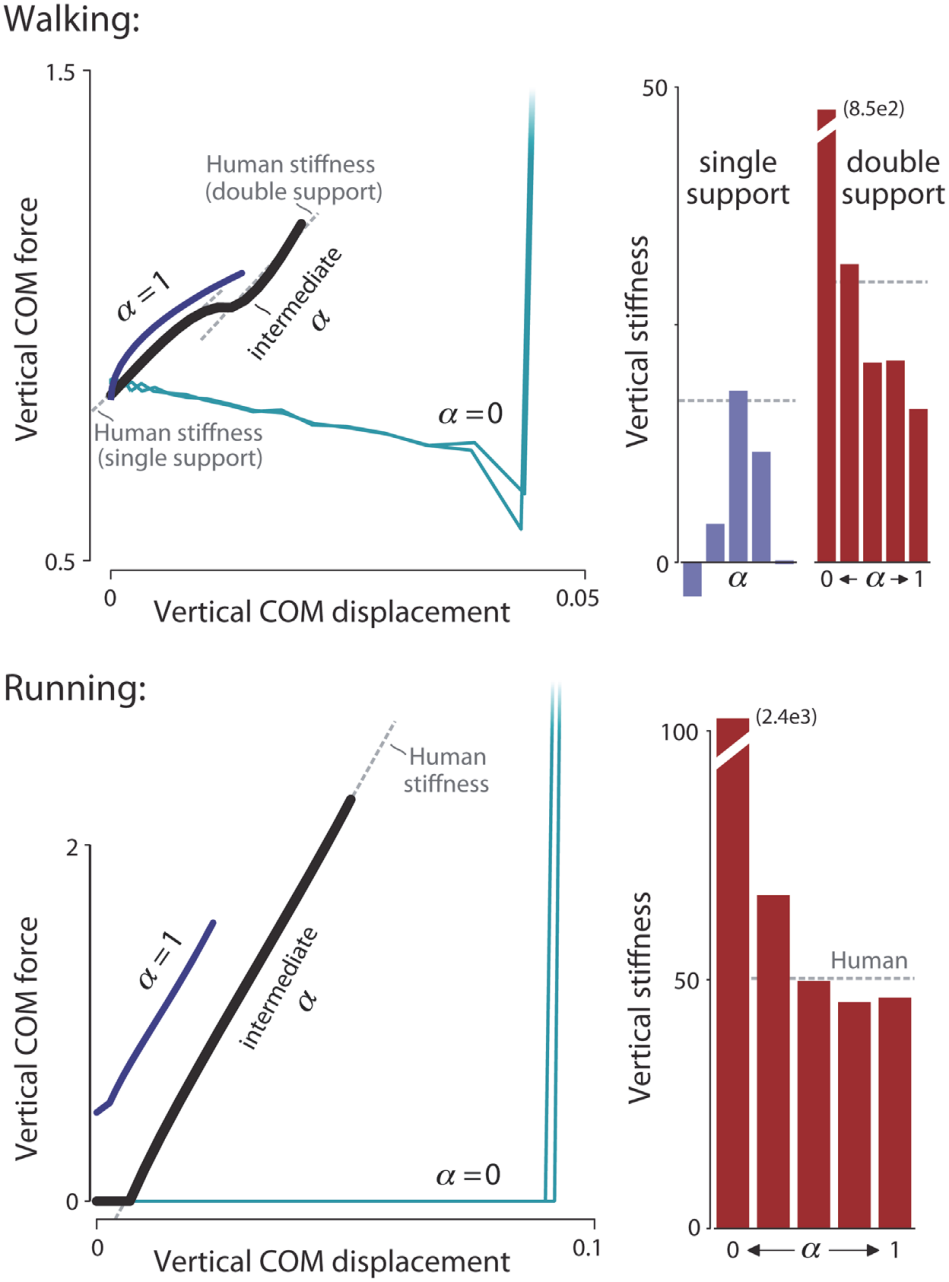
Force vs. displacement (left) and vertical stiffness (right) for optimized gaits. The force vs. displacement relationship shows multiple regions of effective stiffnesses, defined as the slope of the force-displacement curve. Optimization yields walking gaits with two stiffnesses, one relatively low during single support, and one relatively high during double support (with stiffnesses typical of human shown with dotted lines). Fitting line segments to these periods yields estimates of effective vertical stiffness (right) for the model, as a function of weighting coefficient *α*. Model results for intermediate *α* weights are similar to those of human.

We next examine the relationship between total vertical force and vertical center of mass displacement (Fig. 7). Work-minimal walking and running tended toward infinitely stiff behavior, as indicated by the force-displacement slope, during pushoff and collision. With increasing *α*, the smoother walking exhibited lower vertical stiffness during double support, while an intermediate *α* value resulted in a peak single support stiffness. At an intermediate weighting, the model exhibited vertical stiffnesses that qualitatively agree well with stiffnesses fitted from human data. A similar correspondence holds for running. As *α* increased, the ground contact phase exhibited decreasing stiffness, again approximating that of human for an intermediate weighting.

**Fig 8:**
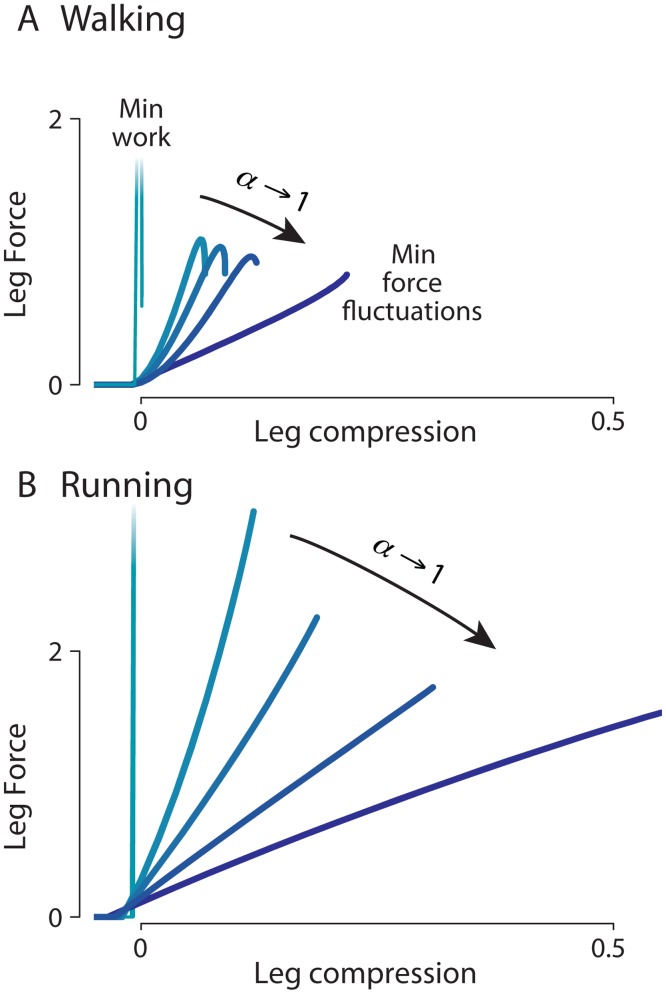
Optimized leg forces vs leg displacements. Work optimized gaits approach infinite leg stiffness (slope of a linear fit to forces) during upward velocity redirection (roughly corresponding to double support in walking, contact phase in running). As the optimization cost includes a larger weighting *α* for force fluctuation, the optimal leg stiffness decreases for both walking and running.

The force-displacement profiles may also be examined more directly in terms of the axial leg forces (Fig. 8). Here it is evident that all of the gaits tended toward a pseudo-elastic behavior [35], with force profiles that exhibited no work loops. Even though arbitrary forces may be obtained, the optimization generally favored profiles that could mostly be produced passively with springs, with one exception: Work-minimizing walking (*α* = 0) requires that the spring store the negative work of collision, and not release it until push-off at the end of stance. Nevertheless, the general tendency is for axial forces to behave with lower pseudo-stiffness as *α* increases.

## Discussion

We had sought to determine whether an objective penalizing leg forces could help explain the rounded profiles for human ground reaction forces. This might improve upon minimization of mechanical work alone [8], which predicts forces with unrealistically high amplitudes at push-off and collision, and unrealistically short double support duration. We found that an opposing extreme is to minimize force alone, which yields walking with an unrealistically long double support phase. But a combination of mechanical work and force costs can produce double-peaked forces and support durations much more similar to humans. In particular, we found that costs based on force fluctuation (i.e. rate of force production) produced the most human-like, rounded force profiles. Below we examine possible implications of this model for human locomotion.

We found it interesting that costs for force amplitude did not favor rounded force profiles. This was true for all amplitude costs (*n* = 0), regardless of the norm exponent *p*. Generally, the higher the exponent, the greater the penalty for high peak force (Figs. 3 and 4). But the optimal means to reduce the peak force while supporting body weight was to increase the surrounding forces for as much time as possible. The tendency was therefore to produce a sharp change in force at the beginning and end of each support phase, and to maintain that phase for as long as possible with a relatively steady force. One exception to this trend was the case of *p* = 1, which simply integrates leg force over time and therefore does not penalize high peaks (Fig. 4, “force amplitude cost”). Although costs for force amplitude may be physiologically justifiable, they do not appear to explain the forces of human locomotion.

Costs including the time-derivative of force performed much better. Examining that cost alone (Fig. 4, “fluctuation force costs”), the tendency was also to avoid high peak forces, but without sharp changes at beginning and end of the support phase. Combining that tendency with the cost for mechanical work resulted in the characteristic rounded double peaks of humans (Fig. 3). Here there were several parameter combinations that could be considered human-like. The simplicity of the present model is surely insufficient to favor any single cost model, but the overall observation is that an optimization cost including both mechanical work and some form of force fluctuation could potentially help to explain human gaits.

It is notable that the optimization yielded some gaits with very little vertical excursion of the center of mass. Once thought to be an energetically economical strategy for human walking [4, 36, 37], experiments show a nearly level COM trajectory to be costly in terms of both metabolic cost and joint work [4]. The present model also finds it to be far from work-minimal, because high smoothness (*α* = 1) requires that the legs produce opposing fore-aft forces, and hence work against each other through most of the stride. In pendulum-like walking (*α* = 0), the legs perform no work except for the instantaneous support transfer, where vertical velocity is actively redirected upwards. This minimizes the time and amount of work of counteracting leg forces, but at the cost of very high forces and force rates (Fig. 5). The addition of a cost for force fluctuation causes the redirection to begin before double support with a preemptive pushoff, and to end after double support when the leading leg completes a smoother collision. This bears closer resemblance to the redirection performed by humans [38]. The optimization illustrates the high cost of low COM excursions, and explains how the human preemptive pushoff phase can satisfy the trade-off between mechanical work and force fluctuation.

The optimization also discovered human-like vertical stiffness properties (Fig. 7). Vertical stiffness summarizes overall oscillatory behavior in humans [39] and a wide range of animals [34]. Here, a cost for work tends toward infinitely high vertical stiffness for both walking and running (Fig. 7), because high stiffness reduces displacement and therefore work. But with an increasing weight on force fluctuation, more displacement is acceptable because it allows decreased peak forces. As a result, the stiffness characteristics become more similar to human, for both single and (in walking) double support. Optimal gaits tended not to favor additional oscillations of force, which can result from relatively stiff legs in elastic models [40]. Here, additional force oscillations entail higher force fluctuation costs, therefore favoring only one or two peaks in ground reaction force.

Another feature of the optimization is that it favors spring-like behavior (Fig. 8), even though the model contains no springs. The spring-like gaits of humans are thought to take advantage of elastic tendons [41], which may reduce the active work required of muscle [42]. But considering that humans are not purely elastic, owing to contractile and dissipative tissues, we find it remarkable that they behave so much like ideal springs. Our model produces similar force profiles despite having no elastic elements. In our mechanical work cost (Equation 2), positive and negative work are weighted equally, neglecting any possible beneficial effect from the addition of actual springs in series with the actuators. While it may be more human-like to include series springs, we believe that the resulting increase in overall efficiency of performing mechanical work would not qualitatively change the tradeoff between the metabolic costs of mechanical work production and muscle force fluctuation. Because the work cost function assumes no spring recovery, the discovery of springlike behavior is more surprising. There are in fact a variety of ways in which spring-like behavior can be optimal without springs [32], but we interpret our findings as possible evidence that pseudo-elastic behavior is economical [35], regardless of actual elastic energy storage capability.

There remains the issue of how a force fluctuation cost relates to physiology. One possibility is that humans favor less impulsive forces or lower peak forces, for example, to avoid pain or discomfort. Such a tradeoff is demonstrated by the strategy humans use when landing from a jump, where they appear to modulate peak forces by bending their knees, even if that strategy costs them additional work to recover their upright stance [43]. It would be challenging to model such subjective preferences quantitatively, but it is possible that such effects apply to locomotion as well.

Another possibility is that force fluctuations are metabolically costly. Indeed, we have observed energetic costs similar to the force fluctuation cost in periodic human motions such as moving the legs back and forth [22, 44] or bouncing vertically about the ankles [23]. Even accounting for work, humans expend additional energy to produce fluctuating forces, perhaps for cyclical activation (see e.g. [45, 46]). Our cost *J*_*f*_ is, however, only a crude representation rather than an accurate model of physiology. A sensitivity analysis suggests that even with variations in the force fluctuation cost’s parameters, it still tends to produce rounded finite force peaks (Fig. 3, “fluctuation force costs”). The optimization is therefore sufficient only to demonstrate the effects of such a metabolic cost, without attempting to model it accurately or for general behaviors. The *α* that yielded the most human-like running gait was comparable in magnitude to that yielding the most human-like walking gait (Fig. 5), suggesting that the cost function roughly generalizes across the two different speeds considered here. We have also found the general effects reported here to apply to a wide range of speeds. However, an optimization approach is unlikely to resolve the cost further without better experimental characterization of the underlying physiology.

Yet another possibility is that the human-like forces are the result of neither cost nor preference, but limitations on body dynamics. The human body contains elastic tendons and deformable tissues [47], and therefore acts as a mechanical filter to reduce force peaks. Humans are also limited in how quickly muscles can produce force, placing a maximum bound on plausible force-derivatives [32]. But just as humans can modulate landing forces, they can also produce quite large peaks simply by landing with straighter knees [43]. Indeed, in informal tests, we find humans to have little difficulty walking more rigidly, given simple instructions to do so (Rebula, 2013; personal observation). It therefore appears that humans could easily produce higher peaks than normally preferred, regardless of the body’s mechanical filtering. Although that filtering is unavoidable to some degree, it also appears insufficient to entirely explain the relatively rounded, low-amplitude forces that humans typically prefer during locomotion. Bounds on force peaks or their derivatives also do not explain the higher metabolic costs associated with faster force fluctuations [22, 23]. Physical limits on force production are undeniable, but they do not appear to explain the human tendency for smooth forces even well below the limits, nor do they explain these empirically observed energetic costs.

There are a number of limitations to this study. We have examined an extremely simple model of locomotion without many anatomical features. Our approach avoided additional parameters that a more complex model would require, and instead focused on the basic requirements of bipedal locomotion. In addition, our cost function also only quantifies production of forces during stance. It does not place a cost on the swing phase, which may be especially unrealistic when the optimization yields a very long double support duration (Fig. 5, “force fluctuation only”) and an instantaneously fast swing. The inertia of the human leg limits movement speed [7, 22, 23], and evidence suggests a substantial metabolic cost for moving the swing leg [22]. That cost could also help to limit the duration of double support, which the present model determines based on stance phase forces alone. Previous findings in penalizing nonlinear force magnitude terms [32] discover similar gaits with an unrealistically short swing phase and reduced force fluctuation. There may also be significant costs for maintaining balance or stabilizing the torso [23, 48], swinging the arms [49], counteracting torques about the vertical axis [49], walking at different step lengths [2] and widths [3] or for cushioning the body [47]. By constraining the nominal walking or running gait, we have effectively treated many such costs as constant. A further analysis may assess the usefulness of the simple cost proposed here to assess a range of gaits with a single choice of *α*. More complex models incorporate additional costs that could contribute to force profiles as well [25–27]. The present study is not intended to exclude other costs, but to suggest that force fluctuations might contribute to human energetic costs, and might even be embedded within the costs of some previous complex models, although to unknown degree. Future muscle studies might reveal whether force fluctuations contribute to empirically derived energetic cost formulas.

There are also limitations on our formulation of the cost of mechanical work. In the human, muscle fascicles perform work in series with elastic tendon, so that the work performed on the leg includes both active and passive contributions [50]. Another issue is that the energetic costs of producing positive and negative work are unequal [6], although our simple cost for work can allow for some of these features through the weighting parameter *α*. Because the net work over a stride is always zero, any relative weighting between positive and negative work is equivalent to an overall scaling of the work cost, which is equivalent to adjusting *α*. Similarly, if a percentage of work is to be treated as elastic (for example 60% [51]), then the active work performed by muscle fascicles is equivalent to a scaling of the work cost *J*_*w*_, and therefore also equivalent to an adjustment to *α*. There may be an advantage to modeling active, passive, positive, and negative work costs more explicitly, but the crude model presented here is a simple means to place a general cost on mechanical work, even if some of the work is performed elastically.

Our model suggests that a cost for force production can potentially help to explain general features of human locomotion. The addition of a cost for force amplitude can reduce the unrealistically high peak forces predicted by a cost for work alone. But we could not produce the rounded, human-like ground reaction profiles unless the cost included a penalty on force fluctuations. A cost for both work and force fluctuations also yielded double support durations and a center of mass trajectory rather similar to those of humans. Here, our model presupposes no pattern for ground reaction forces or kinematics, yet discovers human-like walking and running gaits, driven by hypothetical costs for work and force production alone.

## Supporting Information

S1 Appendix Here we provide details of the constraints used in the optimization. (PDF)
