# Supplementary material for: The Cost of Leg Forces in Bipedal Locomotion: A Simple Optimization Study
Source: PLoS One. 2015 Feb 23;10(2):e0117384. doi: 10.1371/journal.pone.0117384 (PMC4338056; doi:10.1371/journal.pone.0117384)
Supplement: S1 Appendix — (PDF) [file pone.0117384.s001.pdf]

Appendix S1

Here we provide details of the constraints used in the optimization. At evenly distributed times in the trajectory, $i \in [1...N]$, we define body positions ($\vec{p}_i = (x_i, y_i)$), and velocities ($\vec{v}_i = (\dot{x}_i, \dot{y}_i)$), and the force magnitudes along the leading and trailing legs, (f_i and $f_{c,i}$, respectively). We perform an optimization of all of these variables throughout the step,

$$C := \left[\begin{array}{cccccccc} \vec{p}_1 & \vec{v}_1 & f_1 & f_{c,1} & ... & \vec{p}_N & \vec{v}_N & f_N & f_{c,N} \end{array} \right].$$

The optimization routine (a sequential quadratic programming algorithm, the MATLAB fmincon function [33]) searches over C, minimizing the cost function (J) while satisfying the following constraints.

A multiple shooting integration approach is used to constrain the state trajectory to be dynamically feasible. Defining force vectors along the leading and trailing legs as

$$\vec{f}_i := f_i \frac{\vec{p}_i}{||\vec{p}_i||} \text{ and } \vec{f_{c,i}} := f_{c,i} \frac{\vec{p}_i + (L_{\text{step}}, 0)}{||\vec{p}_i + (L_{\text{step}}, 0)||},$$

each state in the trajectory is constrained to be equal to the previous state integrated forward over a time period Δt, ($\Delta t = T_{\text{step}}/(N-1)$), satisfying the differential equations $(\ddot{x}, \ddot{y}) = \vec{f} + \vec{f_c}$, where $\vec{f}$ and $\vec{f_c}$ are linearly interpolated between forces at the control points, $\vec{f_i}$ and $\vec{f_{c,i}}$, respectively. The integration is performed using a fixed step Euler method. To improve the stability of forward integration, we apply 20 intermediate steps between each control time step Δt.

Additionally, the leg force is constrained to be zero at the beginning and end of a stride, $f_1 = 0$, and $f_{c,N} = 0$. The gait is constrained to be a limit cycle of locomotion by constraining the final state of the body mass to be equal to the initial state translated forward by the nominal step length,

$$\begin{bmatrix} x_N + L_{\text{step}} \\ y_N \\ \dot{x}_N \\ \dot{y}_N \end{bmatrix} = \begin{bmatrix} x_1 \\ y_1 \\ \dot{x}_1 \\ \dot{y}_1 \end{bmatrix}$$

Finally, each leg is constrained to exert zero force if the foot is not on the ground (i.e. the distance between the body and the foot's nominal location on the ground is greater than the maximum allowable leg length, 1),

$$\begin{aligned} f_i = 0 &\quad \text{if} \quad ||\vec{p}_i|| > 1, \text{and} \\ f_{c,i} = 0 &\quad \text{if} \quad ||\vec{p}_i + (L_{\text{step}}, 0)|| > 1. \end{aligned}$$

To improve the numerical behavior of the simulation routine, we use an approximate absolute value function when calculating the cost, $|x|_* = \sqrt{x^2 + \epsilon^2} - \epsilon$, with $\epsilon = 1 \cdot 10^{-7}$, which eliminates the first derivative discontinuity of the absolute value function at zero [32]. We found the optimization results were insensitive to small changes in ϵ, since both the absolute value function and this approximate function have a single minimum at zero. With $\epsilon = 0$, the optimization algorithm fails in some cases.

The initial guess for body trajectory was a constant height of 1, a steady forward progression at the nominal speed. The initial guess for leg forces was half body weight with a small amount of added random noise. To ease the optimization problem and test for discretization effects, the optimization was performed at successively higher resolutions (N = 11, 21, and 41), with each optimization providing an initial guess for the next, with a small amount of noise added to avoid local minima. We found no qualitative difference in the resulting forces other than resolution when N was varied from 11 to 41, all results are shown with N = 41. Optimizing a single gait required on average roughly 2 minutes of computation time on computer with an Intel Core i7-2600 3.4 Ghz processor.

The human data are from two subjects, one walking, one running. We believe these data to be representative of previously published data (e.g. [2, 40]).
